# Sky High or Grounded: Nest Site Selection of Herons and Egrets in a Mixed‐Species Colony

**DOI:** 10.1002/ece3.70761

**Published:** 2025-01-01

**Authors:** Farrah Samraoui, Riad Nedjah, Abdennour Boucheker, Hamed A. El‐Serehy, Boudjéma Samraoui

**Affiliations:** ^1^ Laboratoire de Conservation Des Zones Humides University of Guelma Guelma Algeria; ^2^ Department of Ecology University 8 Mai 1945 Guelma Algeria; ^3^ Department of Biology University Badji Mokhtar Annaba Algeria; ^4^ Department of Zoology, College of Science King Saud University Riyadh Saudi Arabia; ^5^ Algerian Academy of Sciences and Technologies El Madania Algeria

**Keywords:** Ardeidae, breeding phenology, colonial Waterbirds, competition, North Africa, resource partitioning

## Abstract

Resource partitioning is crucial for the coexistence of colonial herons, as it allows multiple species to share the same habitat while minimising competition. This study took advantage of a natural experiment in 2006 and 2007 when Black‐crowned Night Herons were prevented from breeding at Lake Fetzara in the first year due to the presence of a feral cat. This event provided valuable insight into the spatial and temporal dynamics of nest site selection among coexisting heron species, which consisted of Cattle Egrets (*Ardea ibis*), Little Egrets ( *Egretta garzetta* ) and Squacco Herons ( *Ardeola ralloides* ). After the cat was removed, egg‐laying began in the core areas of the colony and gradually spread to the periphery. Species that initiated nesting early selected mid‐elevation sites near the tree trunk, which likely offered protection from both ground and aerial predators, while also providing some shielding from solar radiation and strong winds. These early selected sites featured larger branches, which conferred greater nest stability. Vertical stratification was evident among the heron species; however, contrary to long‐standing assumptions, it was not directly related to body size. Both vertical and horizontal stratification were observed, with nests progressively moving higher and further from the tree trunk as the breeding season advanced. The following year, Black‐crowned Night Herons displaced other species to lower heights and positions further from the trunk, highlighting the significant influence of interspecific interactions on nest site selection. This study underscores the complex interplay between nest site selection, biotic interactions and abiotic factors in heron colonies, emphasising the importance of resource partitioning in maintaining species coexistence in densely populated breeding sites.

## Graphical abstract

This study investigates the critical role of resource partitioning in the coexistence of colonial herons, focusing on the spatial and temporal dynamics of nest site selection following a natural experiment at Lake Fetzara in Algeria.

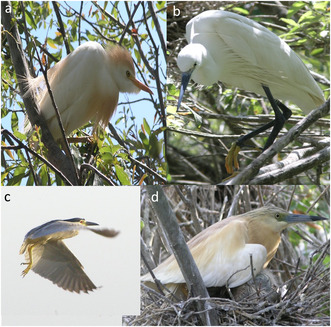

## 1 Introduction

Niche displacement, also known as character displacement, occurs when resources are scarce, leading to the divergence of niches between competing species (Stuart et al. 2014). This process reduces direct competition by promoting the evolution of traits or behaviours that allow species to exploit different resources or habitats (Brown and Wilson 1956). Understanding this concept is fundamental to understanding how species adapt to their environment and minimise competitive pressure. Conversely, when a competitor or predator is eliminated or significantly reduced, an ecological release occurs that allows an organism to expand its niche (Bolnick et al. 2010; Emery and Ackerly 2014). This expansion allows the species to utilise a wider range of resources or habitats that were previously inaccessible due to competition or predators (MacArthur and Wilson 1967; Grant 1986).

Natural experiments provide valuable opportunities to study how coevolution shapes the niches of competitors within ecological communities (Diamond 1983). By observing these naturally occurring scenarios, researchers can gain insights into the dynamics of interactions between species and the evolutionary pressures that drive niche differentiation. Such studies shed light on the complex processes underlying community structure and the adaptive strategies that competing species use to coexist. Connell's seminal work (Connell 1980) illuminated the evolutionary implications of competition, introducing the influential ‘ghost of competition past’. He proposed that historical interactions have driven species adaptations that minimise direct competition in the present, highlighting the importance of experimental methods in studying coevolution and niche partitioning. Building on this foundation, Diamond (1975, 1983) offered further insights into species community structure through his ‘assembly rules’. He argued that species coexistence is governed by niche differentiation and a balance of competitive forces, underscoring, like Connell, the critical roles of competition and coevolution in shaping community structure.

Competition is a fundamental ecological driver of community assembly and a dominant force in evolutionary diversification (Tilman 2004; Ricklefs 2010). The principle of competitive exclusion, a core concept of community ecology, states that species using the same limiting resource can only coexist if they occupy different niches (Hardin 1960; Hutchinson 1961). Density‐dependent habitat selection often mitigates interspecific competition between co‐occurring species (Rosenzweig 1981; Morris 2003; Avgar, Betini, and Fryxell 2020). In heron colonies (heronries), habitat partitioning plays a crucial role in mediating competition for space. In general, vertical stratification within these colonies correlates with body size and time of settlement, with larger species nesting higher and smaller species nesting lower (Burger 1982; Fasola and Alieri 1992; Naugle et al. 1996; Arévalo‐Ayala 2022). The structure of the vegetation also influences this stratification, with the distribution of nesting sites being more pronounced in homogeneous vegetation than in heterogeneous vegetation (Burger 1978; McCrimmon Jr. 1978).

Colonial breeding is a compromise between various advantages and disadvantages (Wittenberger and Hunt 1985). On the one hand, social attraction may facilitate mate choice and provide information on optimal foraging sites (Ward and Zahavi 1973; Custer and Osborn 1978). In addition, a large group may increase safety from predators through rapid detection and collective defence. On the other hand, large aggregations increase aggression and competition for nesting sites, increase susceptibility to parasites and pathogens (Brown and Brown 2004) and make the colony more visible to predators (Danchin and Wagner 1997). While dominance is often correlated with body size (Martin and Ghalambor 2014), aggressive behaviour—a crucial mechanism for space partitioning in herons—can break this size hierarchy. In North America, for example, the invasive Cattle Egret displays greater aggression than native herons and nests higher than expected based on its size. In contrast, as a native species in South Africa, it nests in line with its body length (Burger 1982).

Nest site selection is thought to have crucial effects on fitness, as birds are thought to maximise adult survival and minimise reproductive failure by choosing habitats that protect them and their offspring from predators, bad weather and parasites (Burger and Gochfeld 1985; Martin 1993a, 1993b). However, the study of competition within established communities presents a challenge to field ecologists because these systems are complex and dynamic, often involving intricate relationships between species and environmental factors that are difficult to manipulate and observe in natural settings (Schoener 1983; Gurevitch, Morrison, and Hedges 2000).

This study seeks to explore the nesting phenology and site selection patterns of the Cattle Egret, Little Egret and Squacco Heron, with particular emphasis on assessing the impact of Black‐crowned Night Herons on their nesting preferences (Figure S1). The natural invasion of species such as the Cattle Egret into heronries in North America provided an ideal evolutionary context for the study of competition (Burger 1978). Similarly, the temporary absence of the Black‐crowned Night Heron from the mixed heron colony at Lake Fetzara in 2006, followed by its return in 2007, created a unique natural experiment (Diamond 1983) to examine interspecies competition for nesting sites. Additionally, this research aims to enhance our understanding of how habitat structure influences nesting distribution among herons (Minias 2014) and whether nest site selection depends on specific physical characteristics of nesting placements or on locations within the colony (Minias and Kaczmarek 2013).

## 2 Methods

### 2.1 Study Area

**FIGURE 1:**
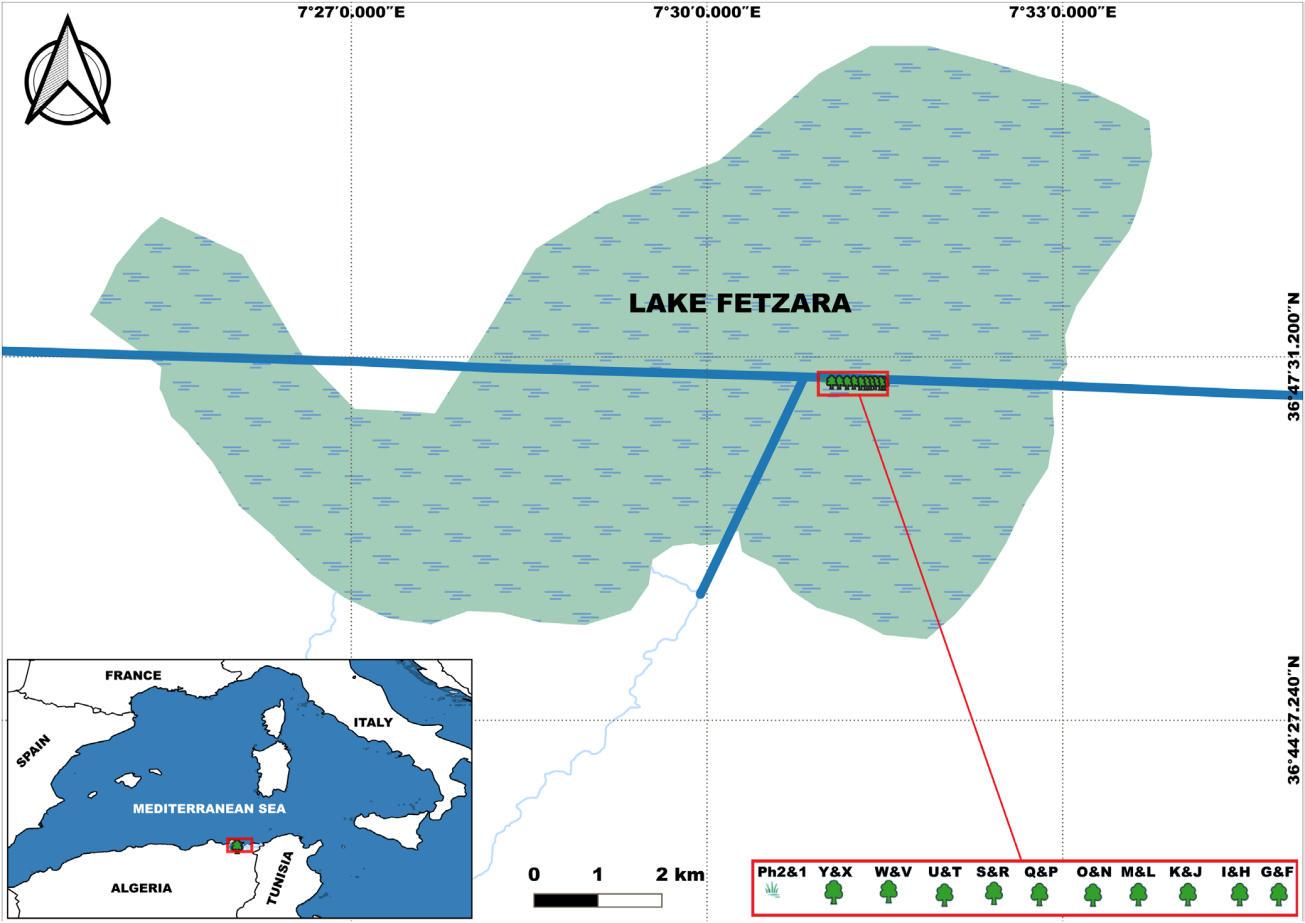
Map of Lake Fetzara depicting the heron colony and associated vegetation. The tamarisk trees ( *Tamarix africana* ), labelled F to Y, are arranged from east to west. Two clusters of common reed ( *Phragmites australis* ) are located at the western end.

Northeastern Algeria is home to the El Kala National Park, a UNESCO Biosphere Reserve that encompasses most of the country's large freshwater and brackish wetlands. These include ponds, shallow lakes, marshes and lagoons, many of which are designated Important Bird Areas and Ramsar sites (Samraoui and Samraoui 2008). Lake Fetzara (36°44′ N, 7°31′ E) is a vast brackish marsh covering 24,000 ha, and dominated by extensive stands of Common Reed ( *Phragmites australis* ), Narrowleaf Cattail ( *Typha angustifolia* ) and Salt Marsh Bulrush ( *Bolboschoenus maritimus* ). The marshland is criss‐crossed by canals, which are mainly lined with Tamarisks ( *Tamarix gallica* ) (Figure 1).

### 2.2 Sampling

The heron colony studied was located on a remnant dyke along a canal that crosses Lake Fetzara (Figure S2). The Tamarisk trees harbouring the colony were aligned along the canal bank and surrounded by reedbeds occupied by nesting Grey Herons and Purple Herons (Nedjah et al. 2010, 2014). The colony was accessed from its eastern end, and each tree was marked from F to Y (trees A to E were never occupied), proceeding from east to west. The position of each tree was plotted on a map. For analysis purposes, individual adjacent trees were lumped into units and the colony was arbitrarily divided into 10 groups of two adjacent units, numbered from east to west (F & G = 1, H & I = 2, and so on, with the western stand of *Phragmites* numbered 11). Every nest was assigned a unique code, and its contents were observed twice a week. Nests were attributed to species by direct observation and identification of the eggs. The date of the first hatching of each brood was recorded using May 1st as Day 1, and the following physical characteristics of nesting placement in the trees were measured: branch diameter (Bdiam, mm), distance from the nest to the tree trunk (Horiz, cm), tree height (Hveg, cm) and nest height above the ground (Hnest, cm). To minimise disturbance to the colony, which can be catastrophic for breeding herons (Tremblay and Ellison 1979), visits were initiated after courtship and early egg‐laying (Frederick and Collopy 1989), and the time spent in each area was strictly limited.

### 2.3 Statistical Analysis

An initial exploration of the data was conducted to identify outliers, zero inflation and collinearity. This preliminary analysis revealed insufficient variation in tree heights, leading to the exclusion of this variable from further analysis. The Shapiro–Wilk test indicated non‐normality, so nonparametric Kruskal–Wallis tests were applied to compare the medians of selected variables—hatching date, nest height, distance from nest to tree trunk, and branch diameter—between heron species. If the Kruskal–Wallis test was significant, post hoc Dunn tests were used for multiple pairwise comparisons.

To assess the influence of tree position and year on hatching date, a generalised additive model (GAM) with an identity link function and a Gaussian error distribution was used, with hatching date as the response variable and tree position (Line) as the explanatory variable. To investigate temporal changes in nest characteristics, a GAM with a Gaussian error distribution and an identity link function was employed, using nest height (Hnest), distance from nest to tree trunk (Horiz) and branch diameter (Bdiam) as response variables, and hatching date, year and species as explanatory variables. To examine the spatial distribution of herons within the heronry, we also modelled the proportion of each species per tree using a GAM with a logit link function and a binomial error distribution, with tree position and year as explanatory variables.

GAM is a generalised linear model in which the relationship between the response variable and the covariates is represented by a smoothing function (Hastie and Tibshirani 1990). This technique offers flexibility in capturing potential nonlinear relationships with covariates. All GAM analyses were performed using the mgcv package, and all statistical analyses were conducted with R (R Development Core Team 2024).

## 3 Results

### 3.1 Phenology of Breeding

**FIGURE 2:**
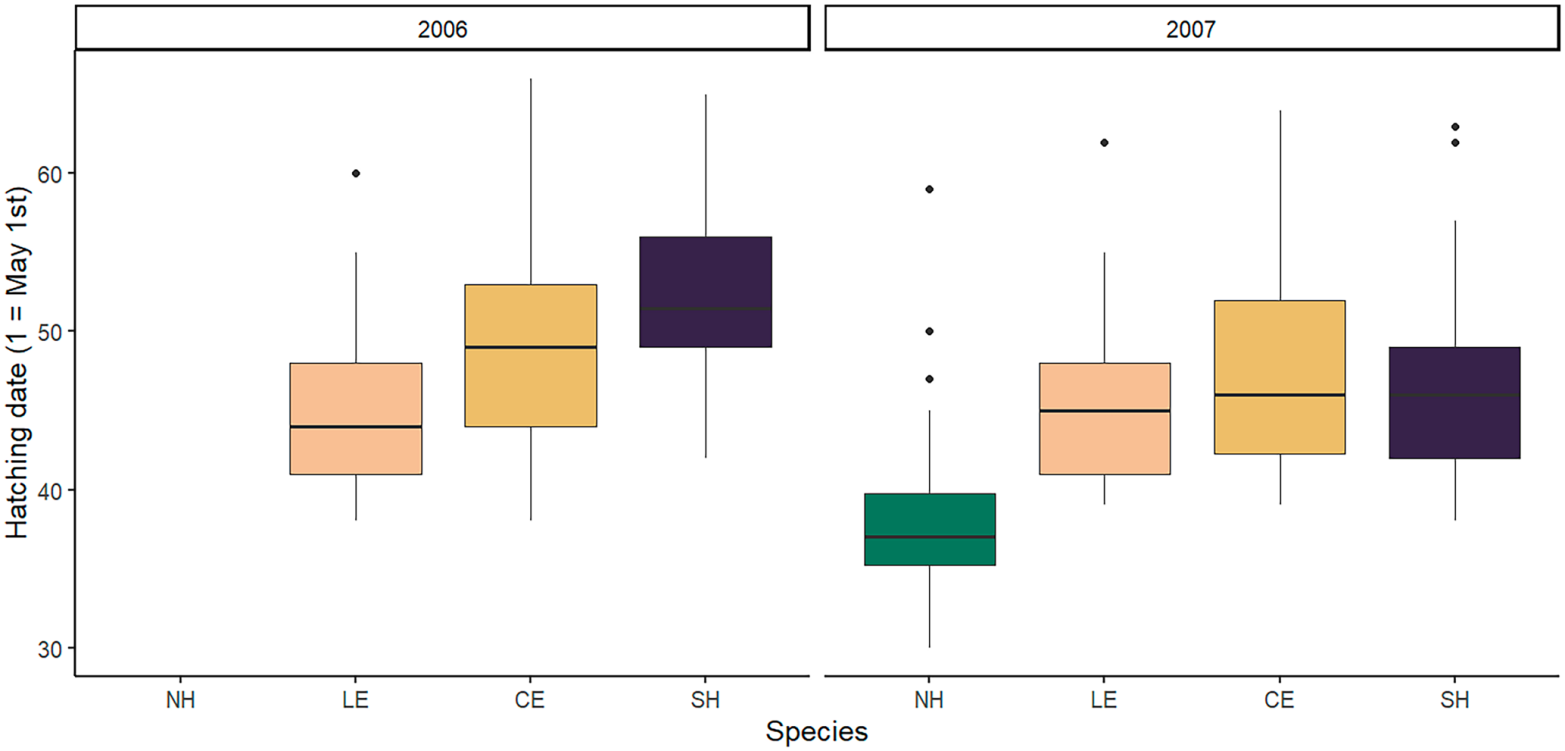
Boxplots of hatching times (using 1 = May 1st) for Cattle Egrets (CE), Little Egrets (LE), Black‐crowned Night Herons (NH; not breeding in the first year) and Squacco Herons (SH) in 2006 and 2007 at Lake Fetzara.

In 2006, a feral cat was found on the dyke where several carcasses of Black‐crowned Night Heron were lying around. After the cat was removed, Little Egret, Cattle Egret and Squacco Heron started laying eggs on the Tamarisk trees along the dyke (Figure 2, Table 1). By then, it was uncertain whether the Black‐crowned Night Heron, prevented from nesting by the cat, had settled elsewhere on the lake or whether they had not bred at all this year. The hatching times of the heron species that bred in 2006 showed a clear temporal separation with significant difference between species (H = 52.5, df = 2, *p* = 4.0e‐12). Little Egret hatched first, at a median time that differed significantly (*p* < 0.001) from that of Cattle Egret, followed by Squacco Heron, which hatched last (*p* < 0.001).

**TABLE 1:** Mean hatching dates (±SD), minimum and maximum values for all species in 2006 and 2007 (using 1 = May 1st).

Species	Year	Mean ± SD	Min	Max	*N*
Cattle Egret	2006	49.5 ± 7.1	38	66	233
Little Egret	2006	44.8 ± 4.6	38	60	77
Squacco Heron	2006	52.4 ± 5.7	42	65	64
Cattle Egret	2007	48.1 ± 6.4	39	64	258
Little Egret	2007	45.5 ± 5.1	39	62	48
Black‐crowned Night Heron	2007	37.9 ± 5.0	30	59	54
Squacco Heron	2007	46.8 ± 5.9	38	63	82

In 2007, the breeding chronology changed significantly, with the average laying date shifting forward (Table 1). Although hatching times were relatively synchronised, there was still a significant difference between them (H = 106.6, df = 3, *p* < 0.001). Black‐crowned Night Heron hatched first (*p* < 0.001 for all three species), followed by Little Egret, which hatched just before Cattle Egret (*p* < 0.04). Squacco Heron hatched last, although their timing did not differ significantly from that of Cattle Egret.

**FIGURE 3:**
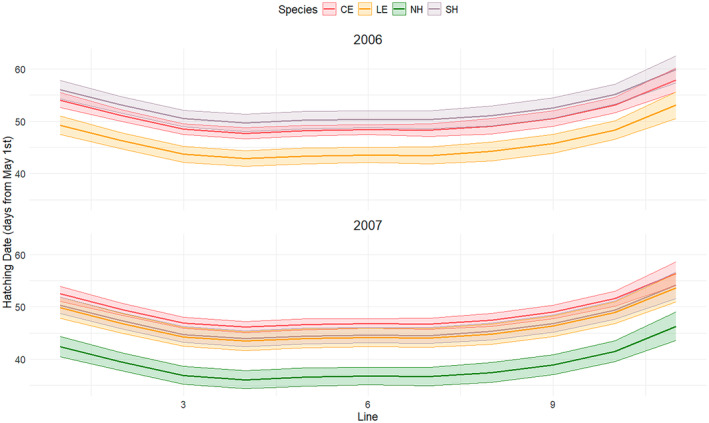
Relationship between hatching date and nest location within the colony. The line represents ten groups of two adjacent trees, numbered from east to west: F & G = 1, H & I = 2, and so on, with the western stand of *Phragmites* numbered 11. Codes: Cattle Egrets (CE), Little Egrets (LE), Black‐crowned Night Herons (NH) and Squacco Herons (SH).

A generalised additive model (GAM) showed a clear spatial pattern, with hatching for all species starting in the central part of the heronry and forming a characteristic U‐shape, indicating delayed nesting at the edges of the heronry (Table 2, Figure 3).

**TABLE 2:** Results of the generalised additive model (GAM) describing hatching date using Line, species and year, as explanatory variables.

Parameter	Estimate	SE	*t*	*p*
Intercept	49.7	0.4	130.3	2.0e‐16
Species_LE	4.8	0.8	6.4	2.6e‐10
Species_NH	10.1	0.9	11.7	2.0e‐16
Species_SH	2.0	0.8	2.5	0.014
Year_2007	1.5	0.5	2.9	0.0042
Species_LE:Year 2007	2.1	1.2	1.8	0.068
Species_SH:Year 2007	−4.2	1.1	−3.9	0.0001
Smooth terms	edf	Ref.df	F	p‐value
s(Line)	5.4	9	15.6	2.0e‐16

*Note:* Smooth Terms: Smooth terms in the model capture nonlinear relationships between explanatory variables (Line) and the response. edf (Effective Degrees of Freedom) indicates the flexibility of the spline fit for Line while Ref.df (Reference Degrees of Freedom) is used for hypothesis testing, comparing the smooth term against a simpler, linear model.

### 3.2 Physical Characteristics of Nesting Locations Nest Height

**FIGURE 4:**
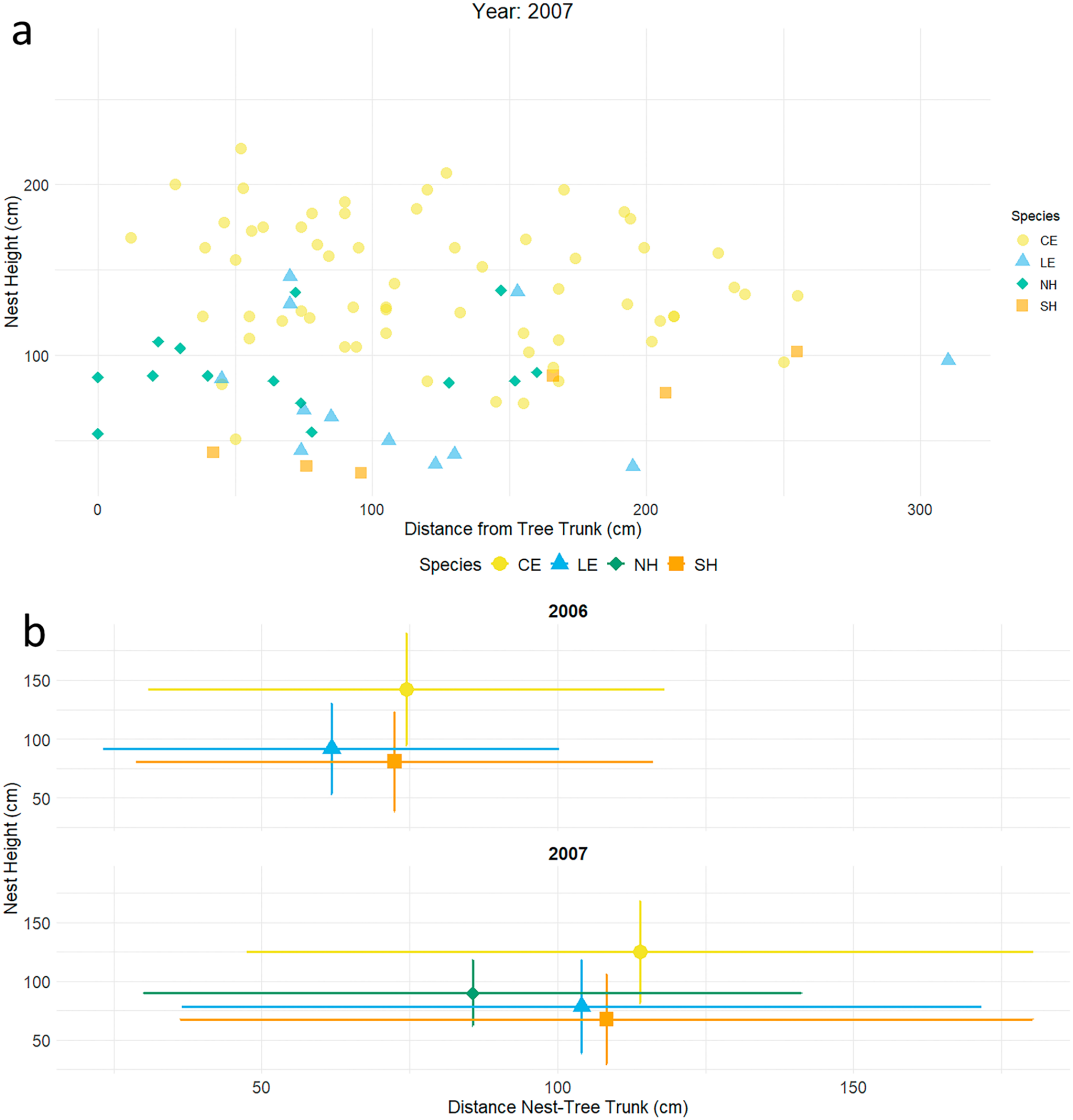
(a) Scatterplot showing the horizontal and vertical placement of nests in four trees (Q0, K, L, P) in 2007. (b) Mean position (nest height vs. distance from nest to tree trunk) for all nests in 2006 and 2007. Gold circles represent Cattle Egrets (CE), green diamonds = Black‐crowned Night Herons (NH), orange squares = Squacco Herons (SH) and blue triangles = Little Egrets (LE).

In both years, all the species overlapped among each other in their nest heights (Figure 4a). In 2006, median nest heights differed significantly among species (H = 128.6, df = 2, *p* < 0.001). Little Egret settled at medium height, followed by Cattle Egret that nested at a significantly higher level (*p* < 0.001). Squacco Heron, the last to settle, placed their nests at lower heights, although the difference with Little Egret was not significant (Table 3).

**TABLE 3:** Mean nest height, distance from nest to tree trunk and branch diameters ±SD for Cattle Egret, Little Egret, Black‐crowned Night Heron and Squacco Heron.

Species	Year	Mean ± SD	Min	Max	*N*
*Nest height (cm)*	2006				
Cattle Egret		142.0 ± 47.4	27	302	233
Little Egret		91.5 ± 38.4	43	232	258
Squacco Heron		80.6 ± 42.3	30	267	64
	2007				
Cattle Egret		125.0 ± 43.1	23	233	258
Little Egret		78.5 ± 39.9	25	165	48
Black‐crowned Night Heron		89.9 ± 27.9	36	165	54
Squacco Heron		67.8 ± 38.1	21	250	82
*Distance nest‐tree trunk (cm)*	2006				
Cattle Egret		74.5 ± 43.7	0	220	233
Little Egret		61.8 ± 38.5	0	395	258
Squacco Heron		72.5 ± 43.7	0	183	64
	2007				
Cattle Egret		114.0 ± 66.5	0	395	258
Little Egret		104.0 ± 67.6	0	310	48
Black‐crowned Night Heron		85.6 ± 55.5	0	300	54
Squacco Heron		108.0 ± 72.0	0	340	82
*Branch diameter (mm)*	2006				
Cattle Egret		36.6 ± 26.2	13	139	233
Little Egret		53.0 ± 37.9	15.9	135	77
Squacco Heron		45.6 ± 42.6	13	155	64
	2007				
Cattle Egret		27.2 ± 8.6	18	113	258
Little Egret		35.3 ± 16.8	24	121	48
Black‐crowned Night Heron		31.2 ± 3.7	25	40	54
Squacco Heron		26.5 ± 3.7	18	38	82

In 2007, a similar pattern of vertical stratification was seen (H = 128.4, df = 3, *p* < 0.001). At medium height, the nest heights of Black‐crowned Night Heron and Little Egret overlapped considerably (*p* > 0.05), and significant differences were only found between Black‐crowned Night Heron and Squacco Heron who settled at lower heights (*p* = 0.006). Cattle Egret again nested significantly higher than all other species (*p* < 0.001), but at lower heights than in the previous year (Figure 4b).

### 3.3 Distance From Nest to Tree Trunk

In both years, all species displayed a broad range of horizontal nest placement (Figure 4b). In 2006, Little Egret, the first to nest, selected sites as close as possible to the tree trunk, while Cattle Egret nested furthest from the trunk. However, no significant differences were found between the species (H = 4.8, df = 2, *p* = 0.09). This pattern changed significantly in 2007 (H = 8.5, df = 3, *p* < 0.04). Black‐crowned Night Heron nested first, closest to the tree trunk, and significantly distant from Cattle Egret, which nested furthest from the trunk (*p* = 0.01). In between, Little Egret and Squacco Heron both nested further away from the trunk, but at no significantly different distance from the other species (Table 3).

### 3.4 Branch Diameter

In 2006, Little Egret nested on the largest branches, Cattle Egret on the smallest branches and Squacco Heron on intermediate‐sized branches. However, there were no significant differences in branch sizes (H = 1.6, df = 2, *p* > 0.05). This pattern changed significantly in 2007 when the early‐breeding species chose the largest branches and the late‐breeding species nested on smaller branches (H = 54.6, df = 3, *p* < 0.001). The branches selected by Black‐crowned Night Heron were larger than those of Cattle Egret (*p* < 0.001) and Squacco Heron (*p* < 0.001). Similarly, Little Egret selected larger branches than those used by Cattle Egret (*p* < 0.001) and Squacco Heron (*p* < 0.001). No other differences were significant (Table 3).

### 3.5 Temporal Variation in Nest Site Characteristics

**FIGURE 5:**
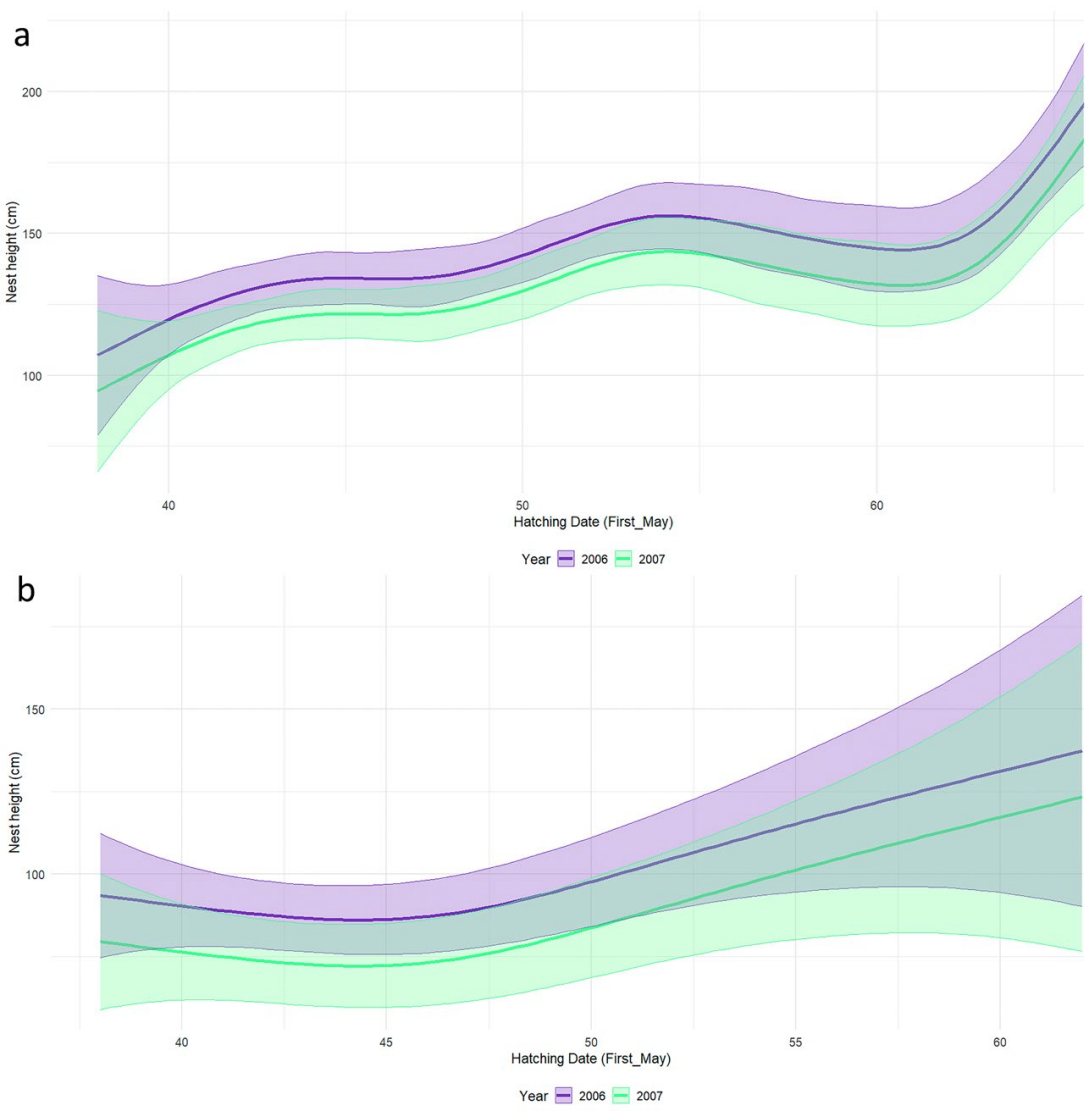
Variation in nest height throughout the breeding period for (a) Cattle Egrets and (b) Little Egrets (using May 1st as Day 1).

**FIGURE 6:**
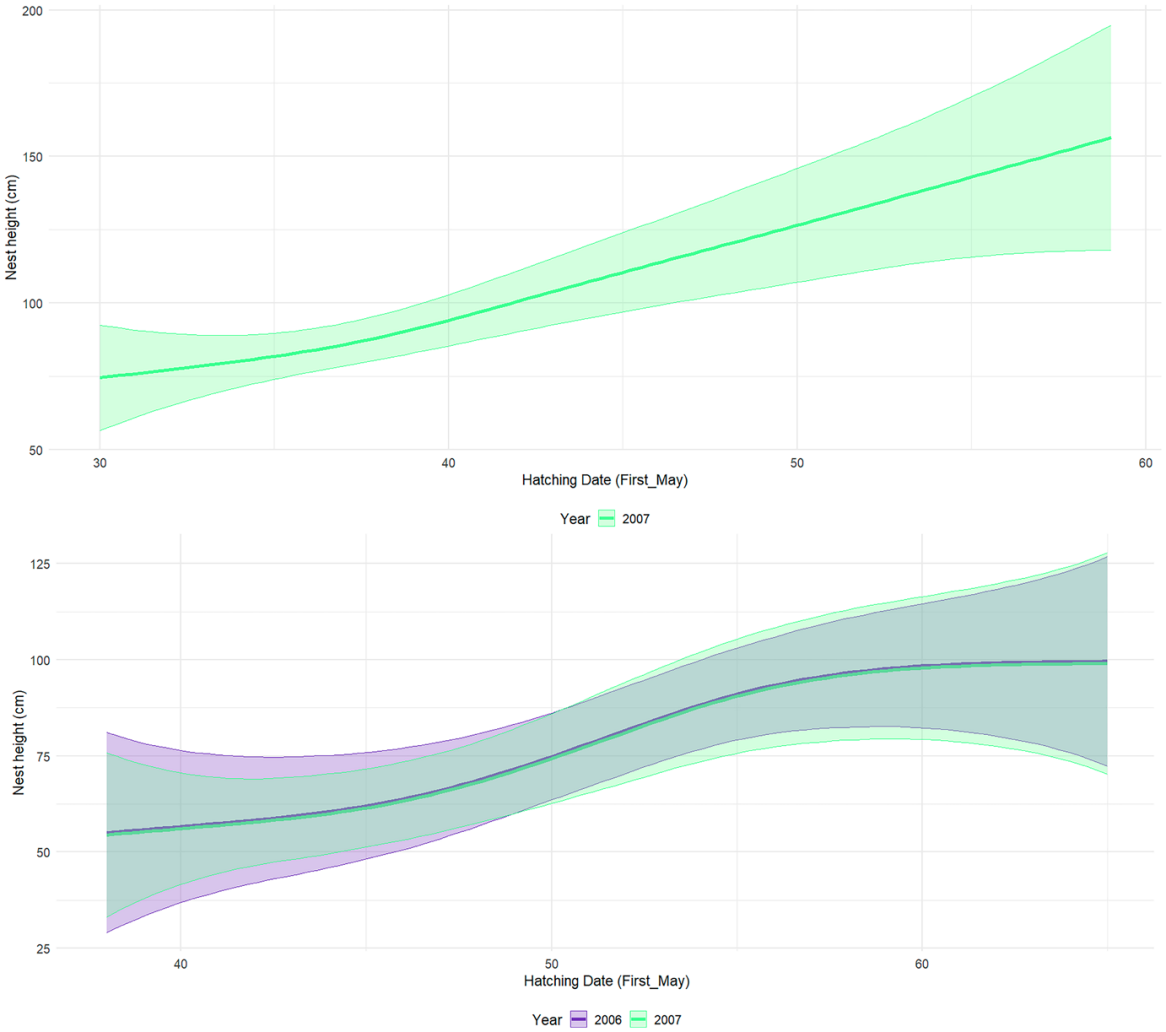
Variation in nest height during the breeding period for (a) Black‐crowned Night Herons and (b) Squacco Herons (using May 1st as Day 1).

**FIGURE 7:**
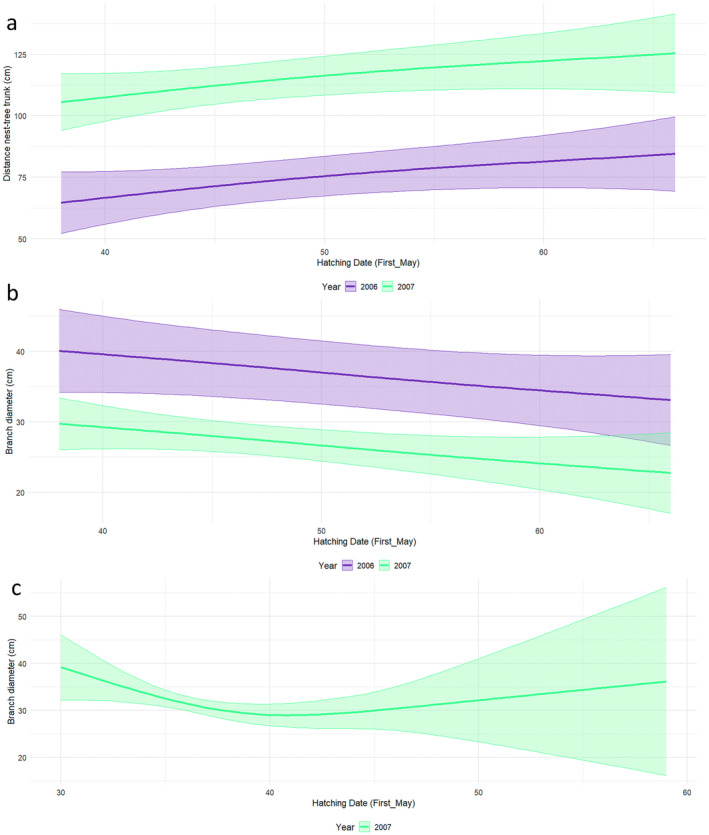
(a) Variation in distance from nest to tree trunk relative to hatching time for Cattle Egrets (using May 1st as Day 1). (b) Variation in branch diameter relative to hatching time for Cattle Egrets. (c) Variation in branch diameter relative to hatching time for Black‐crowned Night Herons.

All four species nested at increasing heights over time (Figures 5 and 6). With time, Cattle Egret increasingly nested further away from the tree trunk (Figure 7a) and on smaller branches (Figure 7b) while Black‐crowned Night Heron also placed their nests on increasingly smaller branches, but greater than 3 cm in diameter (Figure 7c). In contrast, there were no significant changes in the distance to the tree trunk or branch size of the Little Egret and Squacco Heron nests (Table 4).

**TABLE 4:** Results of generalised additive models (GAMs) for nest height, distance from nest to trunk and branch diameter using species, year and hatching date as explanatory variables.

Parameter	Estimate	SE	*t*	*p*
Cattle Egret, nest height				
Intercept	139.9	2.9	48.5	2.0e‐16
Year 2007	−12.5	4	−3.1	0.002
s(Hatching time)	6.2	9	6	2.0e‐16
Cattle Egret, distance nest‐tree trunk				
Intercept	73.7	3.8	19.4	2.0e‐16
Year 2007	40.9	5.3	7.8	5.3e‐14
s(Hatching time)	1	9	0.5	< 0.02
Cattle Egret, branch diameter				
Intercept	37.4	2.2	16.7	< 2.0e‐16
Year 2007	−10.4	2.5	−4.2	3.7e‐05
s(Hatching time)	0.9	9	0.5	0.021
Little Egret, nest height				
Intercept	91.9	4.4	21	< 2.0e‐16
Year 2007	−14	7.1	−2	0.05
s(Hatching time)	2.2	9	0.87	0.018
Little Egret, distance nest‐tree trunk				
Intercept	61.8	6.1	10.1	2.0e‐16
Year 2007	42.3	9.9	4.3	3.7e‐05
s(Hatching time)	9.8e‐11	9	0	0.8
Little Egret, branch diameter				
Intercept	53	6.6	8	4.7e‐10
Year 2007	−17.7	8.1	−2.2	0.03
s(Hatching time)	9.2e‐11	9	0	0.44
Squacco Heron, nest height				
Intercept	73.6	5.1	14.3	2.0e‐16
Year 2007	−0.6	7.1	−0.1	0.94
s(Hatching time)	2.6	9	1.9	2.8e‐04
Squacco Heron, distance nest‐tree trunk				
Intercept	72.5	7.9	9.2	4.3e‐16
Year 2007	35.8	10	3.4	7.9e‐04
s(Hatching time)	1.9e‐10	9	0	0.89
Squacco Heron, branch diameter				
Intercept	45.6	5.3	8.5	5.4e‐12
Year 2007	−19.1	6.3	−3.1	0.0032
s(Hatching time)	3.4e‐11	9	0	0.87
Black‐crowned Night Heron, nest height				
Intercept	89.9	3.2	27.8	2.0e‐16
s(Hatching time)	1.9	9	2.3	6.3e‐05
Black‐crowned Night Heron, distance nest‐tree trunk				
Intercept	85.6	7.4	11.5	5.9e‐16
s(Hatching time)	0.7	9	0	0.11
Black‐crowned Night Heron, branch diameter				
Intercept	31.2	0.7	46.8	2.0e‐16
s(Hatching time)	2	9	0.9	0.03

### 3.6 Species Location in the Heronry

**FIGURE 8:**
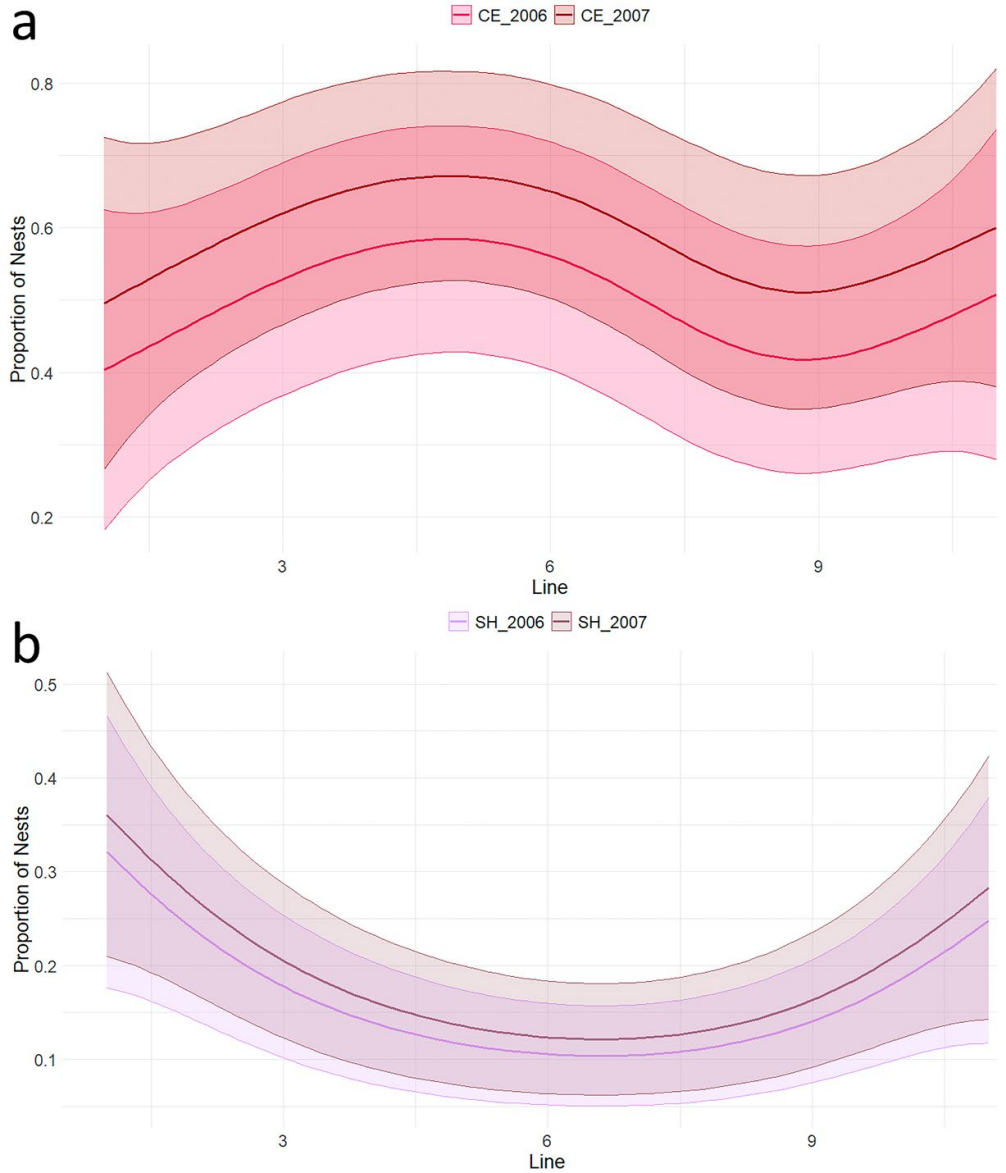
Proportion of nests in relation to location within the colony for (a) Cattle Egrets and (b) Squacco Herons. The line represents ten groups of two adjacent trees, numbered from east to west: F & G = 1, H & I = 2, and so on, with the western stand of *Phragmites* numbered 11. Codes: Cattle Egrets (CE), Little Egrets (LE), Black‐crowned Night Herons (NH) and Squacco Herons (SH).

The proportion of nests for Cattle Egret, Little Egret and Squacco Heron did not vary significantly between years within the colony. However, a clear spatial trend was observed: while the proportion of Cattle Egret was marginally higher close to the central part of the colony and on the western edge of the heronry (Figure 8a), Squacco Heron nested to a considerable extent at the edges of the heronry (Figure 8b; Table 5). No discernible spatial trend could be detected for the Little Egret and Black‐crowned Night Heron.

**TABLE 5:** Results of the generalised additive model (GAM) using proportion of each species as the response variable and year and line as explanatory variables.

Parameter	Estimate	SE	*t*	*p*
Cattle Egret				
Intercept	−0.02	0.2	−0.1	0.9
Year 2007	0.4	0.3	1.2	0.26
s(Line)	2.9	9	0.4	0.28
Little Egret				
Intercept	−1.7	0.2	−8.7	3.2e‐08
Year 2007	−0.5	0.3	−1.5	0.14
s(Line)	0.1	9	0.02	0.21
Black‐crowned Night Heron				
Intercept	−2.8	0.3	−9.5	5.3e‐09
s(Line)	0.4	9	0.1	0.21
*Squacco Heron*				
Intercept	−1.6	0.2	−8	3.0e‐07
Year 2007	0.2	0.3	0.6	0.53
s(Line)	2.3	9	1.5	0.005

## 4 Discussion

### 4.1 Breeding Phenology

The timing of egg‐laying at Lake Fetzara, which typically begins around mid‐May, agrees well with observations from southwest Spain (Parejo, Sánchez, and Avilés 1999), which shares a similar Mediterranean climate. In arid North Africa, the breeding phenology of herons and egrets is strongly influenced by their sensitivity to cold snaps in spring, which can destroy entire colonies or roosts (B.S., unpublished data). In addition, drought stress (Roberts et al. 2021) and high summer temperatures (Gardner et al. 2016) can further affect their breeding success. As observed in Cattle Egret (Samraoui, Menaï, and Samraoui 2007), the laying season tends to be more synchronised and the laying period is significantly shorter. A notable feature of Cattle Egret is the shorter interval between their arrival and the start of egg laying (Burger 1978), which allows them to synchronise with other ardeids. As a result, hatching time, even when adjusted for different incubation periods, may not correlate directly with the timing of site occupancy.

The 2006 natural experiment that prevented Black‐crowned Night‐Heron from nesting at Lake Fetzara emphasises the crucial importance of nesting timing. In contrast to the early but inconsequential nesting of Great Egret in a heronry in North Carolina (McCrimmon Jr. 1978), early nesting of Black‐crowned Night Heron at Lake Fetzara was critical in securing prime nesting sites that would otherwise have been occupied by other heron species. Remarkably, in this region, body size—which is associated with important life‐history traits such as fecundity (Alisauskas 1987; Barbraud et al. 1999) and survival (Price and Grant 1984)—appears to correlate with breeding phenology. Larger species such as Grey Heron and Purple Heron tend to lay their eggs first (Nedjah et al. 2010, 2014), while smaller species such as Cattle Egret and Squacco Heron lay their eggs later.

The timing of egg‐laying was not evenly distributed across the colony but exhibited a characteristic U‐shaped pattern, suggesting that later nests were placed on the periphery of the colony. Central positions within a colony offer better protection from predators, leading to older, presumably higher quality individuals monopolising these core areas (Coulson 1968; Indykiewicz et al. 2019). This spatial pattern has been observed in herons (Hafner 1977; Kazantzidis et al. 1997) and other species (Forster and Phillips 2009), with central nests typically occupied by individuals with higher reproductive success and phenotypic quality.

### 4.2 Physical Characteristics of Nesting Locations

Numerous studies have documented the vertical nest stratification of species within mixed colonies, particularly in heronries with relatively homogeneous vegetation (Jenni 1969). This hierarchical arrangement, where larger species typically nest at higher levels, is often attributed to aggressive interactions related to body size (Burger 1979, 1982; Fasola and Alieri 1992), earlier arrival at colonies (Burger 1978; Burger and Gochfeld 1990) or species composition (Parejo, Sánchez, and Avilés 1999). The vertical alignment observed at Lake Fetzara, where Cattle Egret preferentially occupy the highest vegetation layers, aligns with the findings of Burger (1982) and Hilaluddin, Shah, and Shawl (2003). However, it contradicts earlier studies showing that Black‐crowned Night Heron occupy higher positions in the vertical stratification hierarchy than Little Egret and Cattle Egret (Parejo, Sánchez, and Avilés 1999; Park et al. 2011; Ashoori and Barati 2013). Another contradictory finding is from the Morante colony, where, in the absence of Black‐crowned Night Heron, Little Egret nested higher than Cattle Egret (Parejo, Sánchez, and Avilés 1999).

However, since herons nest in various habitats, including those with heterogeneous vegetation, vertical stratification based on body size is not always evident and considerable vertical overlap between species can occur (McCrimmon Jr. 1978; Beaver, Osborn, and Custer 1980; Park et al. 2011). Several exceptions exist: for instance, Purple Herons have been documented nesting at the lowest levels in mixed heronries in Italy (Fasola and Alieri 1992) and Algeria (unpublished data).

Several studies have noted cases where Cattle Egret nest at heights higher than their body size would suggest, an exception often attributed to their aggressive behaviour (Blaker 1969; Siegfried 1971, 1972; Burger 1978). These patterns of nest site selection reflect the complex interplay between breeding phenology, behaviour with dominance hierarchy rank often correlated with body mass (French and Smith 2005), and habitat structure, illustrating the multi‐layered nature of resource allocation in bird communities. There appears to be a direct competition between Little Egret and Cattle Egret, and between Black‐crowned Night Heron and Great Egret, for overlapping nest sites (Park et al. 2011). Data from 2003 to 2007 across different Algerian wetlands indicate significant overlap in nest site heights among these species and Squacco Heron, but based on minimum and maximum mean heights, Little Egret (30–145 cm) and Squacco Heron (47–153 cm) can still be categorised as low‐nesting species, while Cattle Egret (89–257 cm) and Black‐crowned Night Heron (89–258 cm) are better considered as high‐nesting species (B.S., unpublished data).

At Lake Fetzara, Black‐crowned Night Heron began nesting earlier, but their nesting heights overlapped considerably with those of Little Egret. In contrast, they nested higher than Little Egret in the Axios Delta (Kazantzidis et al. 1997), Camargue (Hafner 1977, 1978; Voisin 1991), Italy (Fasola and Alieri 1992) and Korea (Park et al. 2011). However, this pattern is reversed in Madagascar, where cases of Black‐crowned Night Heron nesting on or near the ground have also been reported (Burger and Gochfeld 1990).

Building nests at higher levels offers several selective advantages, such as better detection of predators, easier access to the nest and avoidance of ground predators (Burger 1979). However, these advantages come with trade‐offs. In North Africa, solar radiation (Touati et al. 2017) and strong dry winds, such as the Sirocco, can significantly affect nesting success (Si Bachir et al. 2008). Nests at higher heights are more exposed to solar radiation (Salaberria et al. 2014), strong winds (Pang, Yu, and Busam 2019) and aerial predators (Frederick et al. 1992; Vennesland and Butler 2004). Consequently, nesting pairs are compelled to seek wind‐protected sites and avoid sun‐exposed areas (Burton 2006).

In addition to vertical stratification, there may also be horizontal alignment in the tree canopy. At Lake Fetzara, nesting herons showed variable positioning in relation to the tree trunk, with Black‐crowned Night Heron nesting closest to the trunk, while Cattle Egret and Squacco Heron nested further out. Snowy Egret also nested further along the branches of trees and bushes in Florida (Jenni 1969). Beaver, Osborn, and Custer (1980) emphasised the importance of nest‐site stability in the selection of a nest site where courtship and mating behaviour takes place and thus the stability of the site is evaluated. This selection pressure also drives nesting pairs to choose robust branches close to the tree trunk that offer more stability against gusts of wind or antagonistic behaviour. Optimal nest site selection is thus influenced by biotic interactions and different environmental conditions (Bennetts et al. 2000), leading to contradictory patterns and suggesting that environmental conditions and colony dynamics may influence nest‐site selection and possibly override the species‐specific tendencies observed in numerous studies. These different selection pressures could explain the contradictory nesting patterns that herons exhibit in different regions.

### 4.3 Temporal Variation in Nest Site Characteristics

The nesting characteristics of the four heron species at Lake Fetzara changed considerably over the course of the breeding season, with all species gradually settling at higher elevations. As the nesting season progressed, Little Egret moved their nests further away from the trunk, while both Cattle Egret and Black‐crowned Night Heron increasingly settled on smaller branches. These results contradict earlier observations by Burger (1982), who assumed that early breeding herons occupy the highest positions in the vegetation. Similarly, a study in Algeria found that the first Cattle Egret to arrive in a monospecific colony chose the highest nesting sites near the tree trunk. However, as the season progressed and more newcomers arrived, the nest height gradually decreased and the distance to the tree trunk increased (Si Bachir et al. 2008). The pattern observed at Lake Fetzara, both with and without the presence of Black‐crowned Night‐Heron, suggests that the most desirable nest sites—those occupied by early breeders—provided sturdy branches close to the tree trunk and at medium height. Overall, nest choice at Lake Fetzara was probably influenced by a combination of biotic factors (such as predation risks, body size and behaviour) and abiotic factors (such as solar radiation and wind).

Remarkable changes were also observed in the characteristics of heron nest sites from year to year. In 2007, Cattle Egret and Little Egret nested at lower heights compared to 2006. In addition, the nests of these species, as well as those of Squacco Heron, were located further away from the tree trunk and on smaller branches. This shift, possibly due to interspecific competition represented by the presence and spatial distribution of Black‐crowned Night Heron, may represent an adaptation to ensure nest stability when species are displaced away from the tree trunk (Beaver, Osborn, and Custer 1980). Similar adaptive behaviours have also been observed in other species: For example, nesting Snowy Egret has been shown to respond to the presence of Grey Heron by moving to lower heights (Burger 1978), and Black‐crowned Night Heron showed a similar response when cohabiting with Grey Heron (Fasola and Alieri 1992).

### 4.4 Species Location in the Heronry

The centre of bird colonies is typically characterised by low predation risk and high reproductive success (Hafner 1978), often leading to the under‐representation or exclusion of less competitive species in these prime areas. At Lake Fetzara, the smallest colonial ardeid, the Squacco Heron, exhibited similar nesting behaviour to the Snowy Egret, opting to nest closer to the periphery of the heronry compared to the Cattle Egret (McCrimmon Jr. 1978). In a comparable heronry in South Carolina, Great Egret and Cattle Egret demonstrated distinct differences in the distribution of their nests within the colony, indicating clear horizontal zonation. This pattern is consistent with earlier observations by Jenni (1969), who reported similar spatial distribution behaviour in Snowy Egret.

These findings highlight the importance of microhabitats and spatial partitioning within heronries, where species display both vertical stratification and horizontal differentiation in nest site selection. Such zonation likely reduces interspecific competition and promotes coexistence by enabling species to utilise different microhabitats within the same colony (Burger 1978; Ando 1993; Park et al. 2011). It is noteworthy that Squacco Heron prefer to nest in reedbeds (Kazantzidis, Yfantis, and Poirazidis 2013), suggesting that Tamarisk trees may not be an optimal habitat for this species. This complex nesting behaviour illustrates the adaptive strategies employed by herons and egrets to optimise reproductive success and minimise competitive interactions.

### 4.5 Niche Partitioning Among North African Colonial Herons and Egrets

As a marsh species (Kushlan and Hancock 2005), Purple Heron may nest alongside other colonial ardeids, but they are mainly restricted to the large reedbeds of Lake Fetzara (Nedjah et al. 2010). They generally do not compete for nesting sites as they are among the first to occupy breeding areas and usually nesting at lower heights than later arriving species. The situation is similar for the Grey Heron, a rare breeder in Algeria (Samraoui et al. 2011). Although Grey Heron may nest in mixed heronries at greater heights than Purple Heron (Nedjah et al. 2014), they are also largely confined to reedbeds in Lake Fetzara.

The partitioning of resources among nesting herons is also reflected in their food choices, which likely reduces competition. Analysis of the food boluses from six species (Purple Heron, Black‐crowned Night Heron, Glossy Ibis, Little Egret, Squacco Heron and Cattle Egret) revealed a clear relationship between prey size and heron size, with larger herons consuming larger prey and smaller herons selecting smaller prey (Samraoui et al. 2012). In addition to prey type and size, patterns of resource utilisation suggest that herons and ibises nesting side by side also partition their resources according to the timing of breeding and foraging microhabitats. This partitioning minimises direct competition and facilitates coexistence (Samraoui et al. 2012). These results are consistent with established ecological theories that propose niche differentiation as a mechanism to reduce competition and promote coexistence of species through differential resource utilisation (Schoener 1974; Connell 1980).

The timing of nest establishment, social dominance systems, nest density and environmental parameters are critical factors influencing nest site selection and reproductive success in birds (Jenni 1969; Frederick and Collopy 1989; Naugle et al. 1996; Parejo, Sánchez, and Avilés 1999). Nest site selection is particularly important in situations of intense predation or when environmental factors limit the availability and number of safe, sheltered locations (Frederick and Collopy 1989). Various trade‐offs are likely to be involved in this process (Fisher and Wiebe 2005; Minias and Kaczmarek 2013). In habitats with heterogeneous and low vegetation, vertical stratification patterns often decrease because the lack of tall structures forces birds to nest at similar heights, thereby reducing the typical vertical differentiation observed in areas with more uniform and taller vegetation (Burger 1979; Burger and Gochfeld 1990). Furthermore, environmental factors such as harsh weather conditions (Chesson and Huntly 1997) and predation (Roper 2003) can further exacerbate this lack of stratification by modulating the intensity of interspecific and intraspecific interactions.

## Author Contributions

**Farrah Samraoui:** conceptualization (equal), investigation (equal), project administration (lead), resources (lead), writing – review and editing (equal). **Riad Nedjah:** investigation (equal), writing – review and editing (equal). **Abdennour Boucheker:** investigation (equal), writing – review and editing (equal). **Hamed A. El‐Serehy:** funding acquisition (equal), writing – review and editing (equal). **Boudjéma Samraoui:** conceptualization (equal), formal analysis (equal), methodology (equal), supervision (equal), visualization (equal), writing – original draft (equal).

## Ethics Statement

This study was approved by the M.E.S.R.S. and all procedures followed were in accordance with international ethical standards.

## Conflicts of Interest

The authors declare no conflicts of interest.

## Supporting information


**Figure S1.** Photographs of: (a) Cattle Egret, (b) Little Egret, (c) Black‐crowned Night Heron and (c) Squacco Heron.

**Figure S2.** A view of the heron colony located along the main canal at Lake Fetzara.

## Data Availability

Data are available at: https://datadryad.org/stash/share/‐9Y9vXuPOy‐P7_SonOqeayTd3Q3xQ_I‐tGFeVnIph6g.
