# Supplementary figures and images for: Sky High or Grounded: Nest Site Selection of Herons and Egrets in a Mixed‐Species Colony
Source: Ecol Evol. 2025 Jan 1;15(1):e70761. doi: 10.1002/ece3.70761 (PMC11693640; doi:10.1002/ece3.70761)

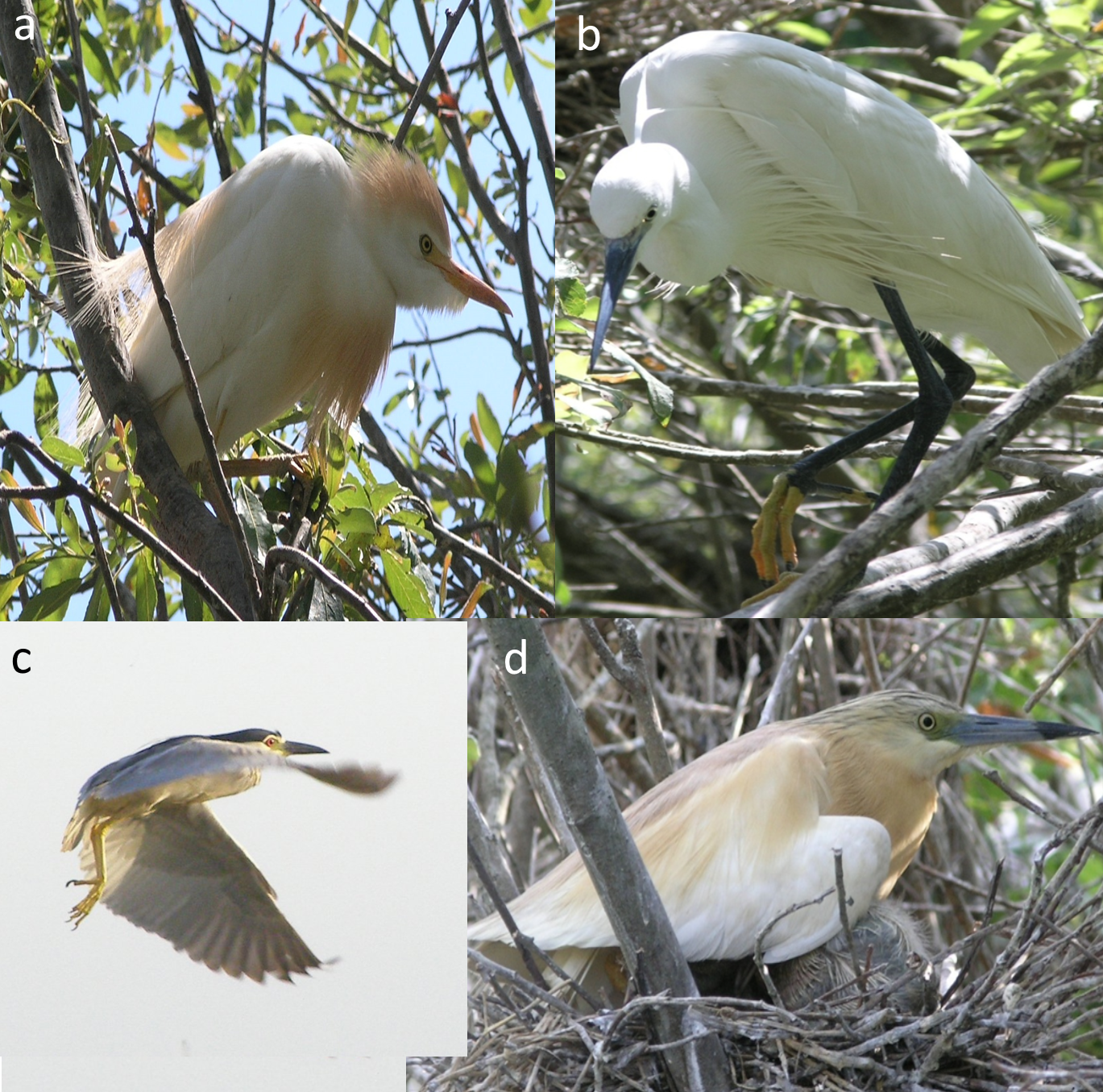

Supplement: Supplementary file 1 — Figure S1. Photographs of: (a) Cattle Egret, (b) Little Egret, (c) Black‐crowned Night Heron and (c) Squacco Heron. [file ECE3-15-e70761-s002.tiff]

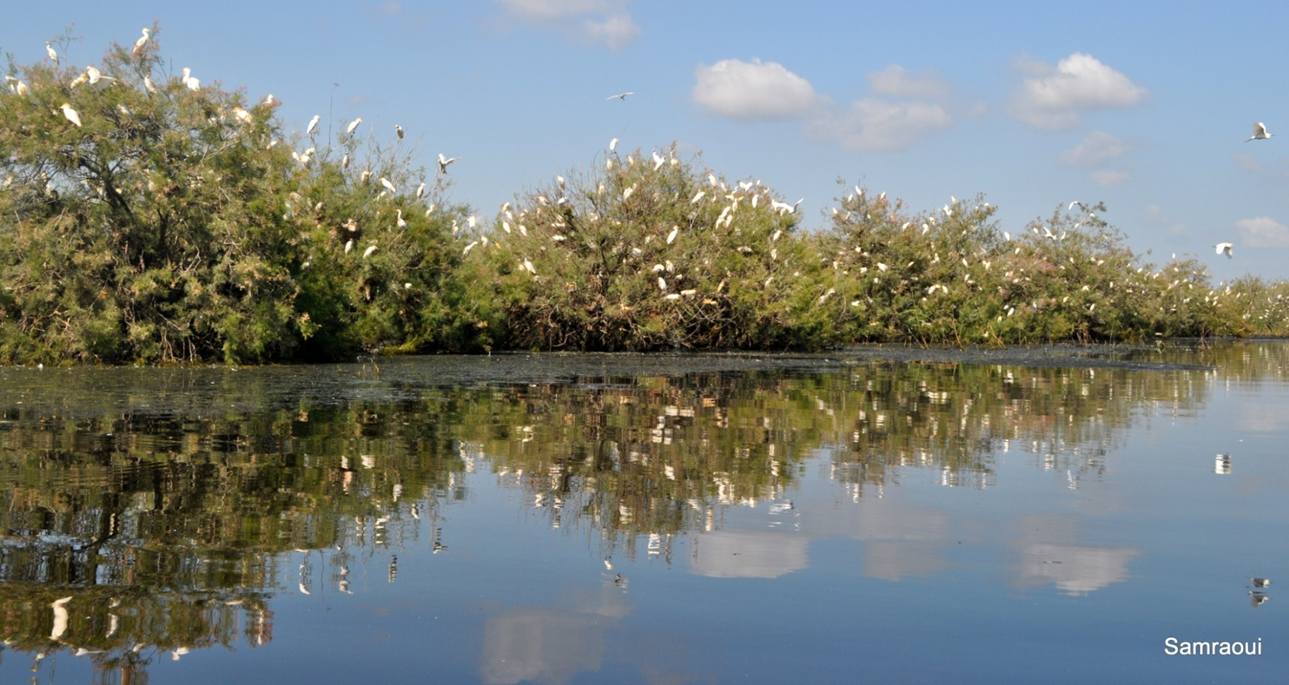

Supplement: Supplementary file 2 — Figure S2. A view of the heron colony located along the main canal at Lake Fetzara. [file ECE3-15-e70761-s001.png]
